# Performance comparison of instrument automation pipelines using different programming languages

**DOI:** 10.1038/s41598-023-45849-y

**Published:** 2023-10-30

**Authors:** Ankur Kumar, Mayank Goswami

**Affiliations:** Divyadrishti Imaging Laboratory, Department of Physics, IIT Roorkee, Haridwar, Uttarakhand India

**Keywords:** Engineering, Mathematics and computing

## Abstract

The article presents a performance analysis of fully automated, in-house developed 2D ultrasound computerized tomography systems using different programming languages. The system is fully automated in four programming languages: LabVIEW, MATLAB, C and Python. It includes codes for sensors, instruments interfacing, real-time control, synchronized data acquisition, simultaneous raw data processing and analysis. Launch performance, eight performance indices and runtime performance are used for the analysis. It is found that C utilizes the least processing power and executes fewer I/O processes to perform the same task. In runtime analysis (data acquisition and real-time control), LabVIEW (365.69 s) performed best in comparison to MATLAB (623.83 s), Python (1505.54 s), and C (1252.03 s) to complete the experiment without data processing. However, in the experiment with data processing, MATLAB (640.33 s) performed best in comparison to LabVIEW (731.91 s), Python (1520.01 s) and C (1930.15 s). Python performed better in establishing faster interfacing and RAM usage. The study provides a methodology to select optimal programming languages for instrument automation-related aspects to optimize the available resources.

## Introduction

Instrumentation and automation have always supported the development of science & research for industrial growth. Generally, automation requires interfacing of various instruments, sensors, and electromechanical components to work in coordination and perform predefined tasks with fewer manual interventions. Automation provides the convenience of performing various monotonous tasks quickly and repeatedly with relatively better precision and accuracy. With the advancement of microcontrollers, integrated circuits, sensors, and electronics, the development of custom-designed systems for scientific research is increasing^1^. Automation of any system requires developing multiple sets of algorithms to perform predefined tasks in a synchronized fashion. The task can be related to triggering various sensors, devices, actuators, or data processing to analyze the acquired data. Programming languages provide the framework to implement these set of algorithms. Programming languages play an extensive role in computation, control systems, instrumentation, device development, and automation^2–7^. Conventionally, the choice of these languages depends upon the developer’s level of comfort and may not be optimal as far as the performance of the overall system is concerned. While developing a fully automated ultrasound computed tomography (UCT) system, we faced the dilemma of whether we would be using the optimal programming language, otherwise costing us the overall performance.

Optimal performance and efficiency are the basic requirements of data-intensive and complex systems. Sometimes, the signal data must be analyzed firsthand before controlling the mechanical components, that requires a faster analysis tool to perform control and coordination in real-time. A better programming language implementation can make a system efficient and improve performance. Performance evaluation of programming languages for instrument automation is one of the least explored research topics. In literature, programming languages are compared for different fields of applications. In^8^, analysis is performed to conduct an empirical study to analyse the productivity variations across different programming languages. In bioinformatics, programming languages are compared for a full-fledged next-generation sequencing tool^9^ and three standard bioinformatics methods^10^. In^11^, three high-performance programming languages are compared for parallel metaheuristics. In^12^, programming languages are compared for computationally intensive next-generation astrodynamics systems. Several programming languages are compared in macroeconomics to solve stochastic models^13^. The criteria for the selection of programming languages may include a convenient user interface, richness in an already developed function library, runtime performance, efficiency in repeatability, support forums, compactness in the length of codes, RAM, memory, and processor utilization^8,14–16^. The performance of the programming languages has been studied in the various aspects of instrument interfacing, data acquisition, instrument control, and data processing^14,17^. The existing studies are primarily focused on the computation point of view without including the different aspects of automation.

To choose the best option for in-house developed UCT automation, we have compared the performance of two commercially available programming languages, namely: LabVIEW™, MATLAB®, and two general-purpose computer programming languages: Python and C^14,18–20^. The comparison is carried out by designing multiple experiments to perform data acquisition, reading/writing data, data processing, and real-time control, individually and collectively. The performance parameters are (a) launch performance, (b) performance indices, (c) runtime performance.

### Motivation

Automation of a system includes various aspects such as components interfacing, data acquisition, processing, electromechanical controls, etc. Generally, research groups just go for the programming languages they are familiar with, resulting in limited efficiency and sub-optimal performance. It may also affect the quality of the output results. Articles that focus on the performance evaluation of multiple automation tools for a given system are rare. To the best of our knowledge, we could not find an article in the literature that evaluate the programming languages’ performances for instrumentation and automation related aspects. In this article, we seek to fill this gap by providing a methodology to analyse and compare the performance of programming languages from automation perspective.

## Methodology

### Brief details about the UCT system

**Figure 1:**
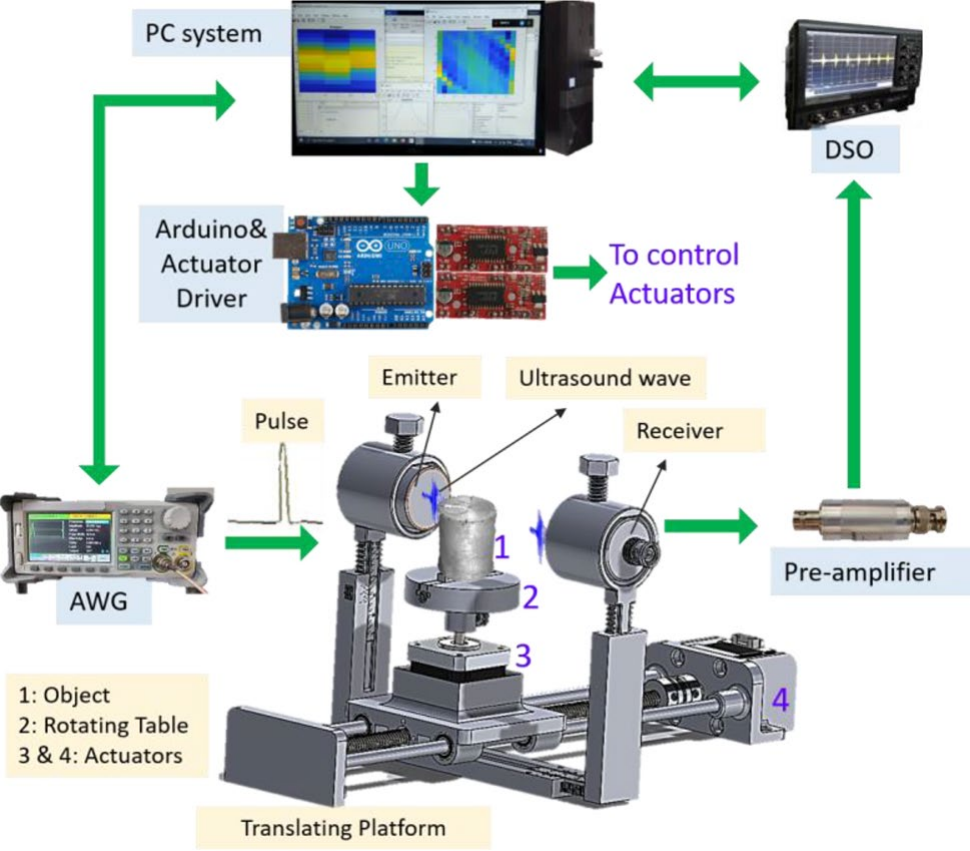
The automated UCT system.

The automated UCT system comprises two non-contact ultrasound (NCU) transducers, an arbitrary wave generator (AWG), a digital storage oscilloscope (DSO), a microcontroller, electromechanical components, and a processing system, as shown in Fig. 1. The AWG, DSO, and microcontroller are connected to the PC via a USB port. The AWG is coupled to one ultrasound transducer, while DSO is coupled to another via BNC cables. A cheap but reliable microcontroller Arduino UNO R3 is used to control actuators in real-time. The performance of programming languages, however, is independent of the choice of any microcontroller. The microcontroller is connected to the actuators via driver electronics based on an H-bridge circuit to precisely control the motor’s shaft. It also provides the convenience of setting micro-stepping resolution as per the requirement. The electromechanical components consist of the rotating table and the linear translation platform coupled with linear ball bearings and a threaded-rod system.

The transducers are placed into their respective stationary 3D printed holders on this platform. The object (to be scanned) is placed on the rotating and sliding table. In our design, AWG generates an input pulse to trigger one ultrasound transducer (called emitter henceforth). In turn, the transducer produces ultrasound waves that traverse through the object and detected by the other transducer (termed as receiver) placed at the other end of the object. The DSO, coupled with the receiver, acquires the raw data and performs necessary analog to digital conversion with a high sampling frequency of 10 × 10^9^ Samples/s (GS/s). The parallel beam geometry is applied to acquire and process the scanning data. The process is repeated multiple times while linearly translating and rotating either the transducer pair or object to reduce the data sparsity. The acquired data is processed to reconstruct the profile of the specimen.

The automation codes are executed on the same PC. The Dell Inspiron Desktop computer equipped with an i5 6400 processor, and 8 GB RAM is used. The system is installed with the Windows 10 (21H2) 64-bit operating system. The same algorithms are implemented to develop the programming codes from scratch to minimize the difference and keep the analysis consistent. The present methodology is generic, and its OS dependent performance can be evaluated.

#### Instrument interfacing

The devices and instruments can be connected to the PC through USB Test and Measurement Class (USBTMC), Serial Communication, Wi-Fi, Ethernet, IEEE 488.2I (GPIB), etc. VISA (Virtual Instrument Software Architecture), an instrument driver, is installed on the PC to facilitate communication. VISA is a standard for configuring, programming, and troubleshooting instruments comprising GPIB, VXI, PXI, serial (RS232/RS485), Ethernet/LXI, and/or USB interfaces^21,22^. It includes utilities and low-level control features required to control the instruments. The AWG and DSO are connected to the system via the USBTMC interface. In DSO, the connection via USBTMC is configured by selecting the utilities > setup > Remote > USBTMC in the DSO. The AWG has a plug-and-play interface and does not require any setting selection. The microcontroller is controlled through a UART (Universal Asynchronous Receiver Transmitter) serial communication interface via a USB connection^23,24^.

The instruments can be interfaced with PC using programming languages either by developing custom algorithms or by using the instrument drivers provided by the manufacturers^25,26^. These drivers offer convenience in controlling and automating the instrument by translating the algorithms into Standard Commands for Programmable Instrumentation (SCPI) commands that the instrument can understand^27–29^. These drivers provide the utility for converting received raw data into a readable numeric format. In our case, these drivers are available for MATLAB and LabVIEW only by AWG and DSO manufacturer support websites. So, custom algorithms for input/output operations using SCPI commands are developed for automation for C and Python. These custom algorithms are developed using instrument commands for input–output operations and implementing the data conversion algorithms. The availability of these drivers and interfacing types used to establish the connection for the respective languages is tabulated in Table 1. The Arduino is controlled using a serial communication interface. The primary control code is embedded into the Arduino, which is called by a secondary code segment that can be written in different languages. It provides stability in real-time control. Whenever the coded instructions are sent from the programming language to the Arduino, it decodes them into a set of instructions, then controls the actuators accordingly. Arduino IDE (version 1.8.19) is used to write and deploy the serial interfacing code. It is ensured that all IDEs have no cross dependencies.

**Table 1:** Interface type and driver availability.

Programming languages	IDE	Instrument	Interface type (USB Based)	Driver/firmware availability
C	Visual Studio (VS) v17.00 *Compiler*: gcc 10.3.0 (Rev5, Built by MSYS2 project)	AWG	USBTMC	Custom designed
		DSO	USBTMC	Custom designed
		Arduino	Serial Communication	Custom designed
Python	Python 3.9.6 (Spyder)	AWG	USBTMC	Yes
		DSO	USBTMC	Custom designed
		Arduino	Serial Communication	Custom designed
MATLAB	MATLAB R2019b	AWG	USBTMC	Yes
		DSO	USBTMC	Lecroy_basic_hr_driver
		Arduino	Serial Communication	Custom designed
LabVIEW	LabVIEW 2018	AWG	USBTMC	SDGX LabVIEW Driver 1.0.1
		DSO	USBTMC	IVI Driver 3.2.9.0 × 64, LeCroy VICP Passport
		Arduino	Serial Communication	LIFA_base

**Table 2:**
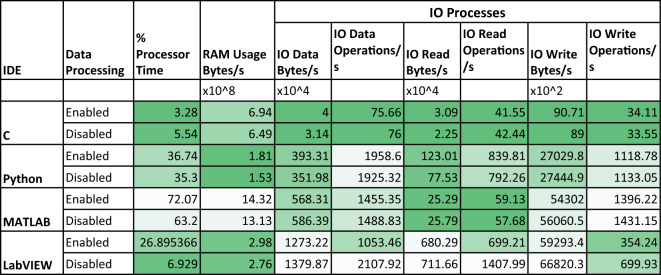
Mean value of parameters used for performance analysis.

**Table 3:** Hardware configuration details.

	Hardware 1	Hardware 2
Processor	i5 6400	*AMD A6 PRO-7400B R5*
RAM	8 GB	16 GB
Motherboard	0J4NFW (A01)	A78F2P – M2/V1.0 (P21-A3E)
OS	*Windows 10 (Version 21H2)*	*Windows 10 (Version 22H2)*
External GPU	*Absent*	*Absent*
Integrated GPU	*Intel HD 530*	*AMD Radeon R5*

#### Data acquisition

The 12-bit raw data is acquired while scanning the object by varying the number of detectors and projections. The acquired data is formatted and written to a “.txt” file. The size of these files depends on the sampling rate, number of detectors and projections. Acquired data file contains time and amplitude data of the signal. The study is carried out to analyze the performance in data acquisition, reading and writing the acquired data with variations in data size.

#### Data processing

It includes raw data processing, signal processing, generating graphics, representing the processed data, and performing several read/write operations. Data processing mainly involves the algorithms to process the ultrasound signal to extract meaningful data called projection data^30^. Projection data is then used to reconstruct the object’s profile via a separate reconstruction algorithm.

**Figure 2:**
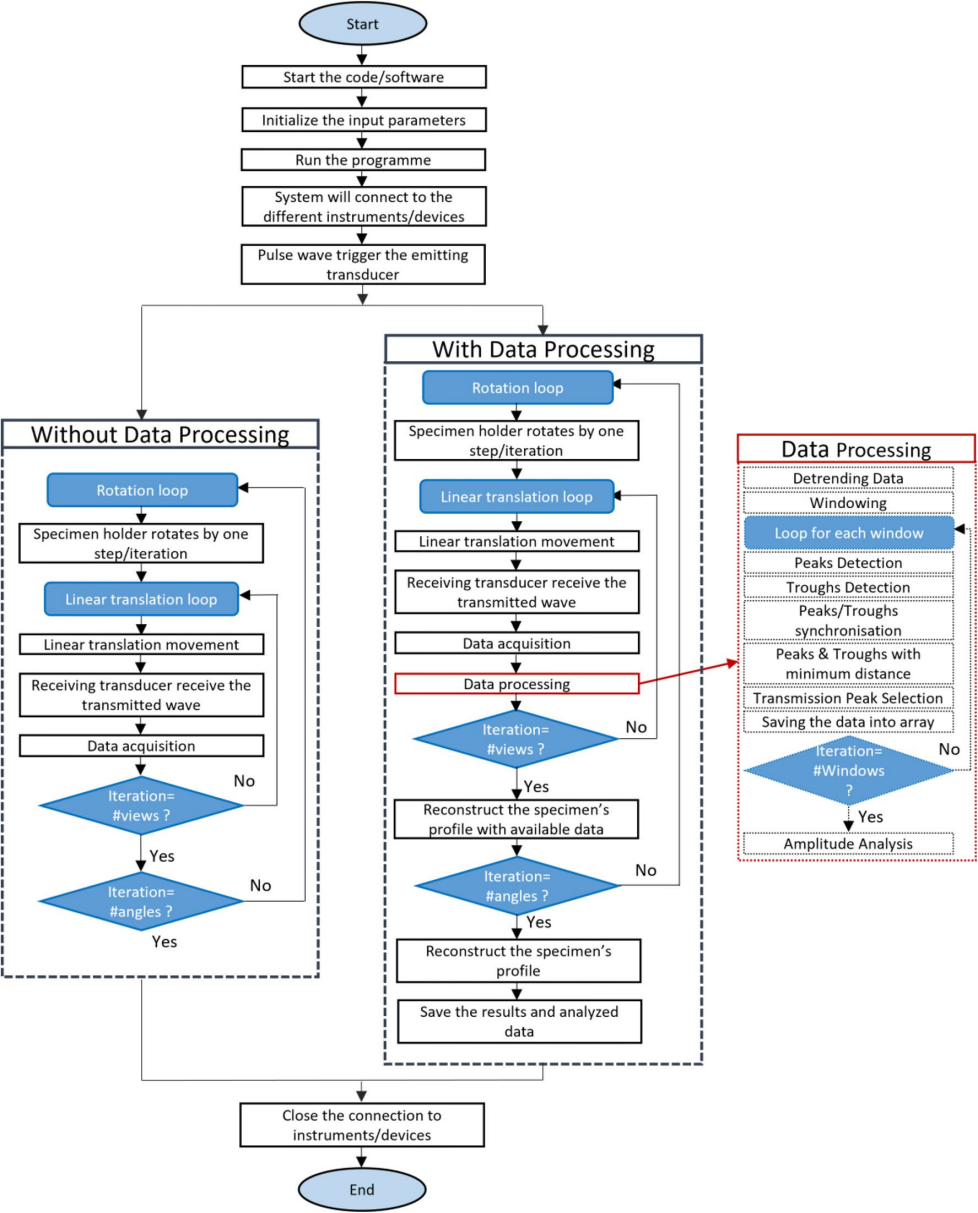
Process flow diagram.

The analysis is carried out for two different classes: (a) without signal processing and (b) with signal processing. The process flow diagram is shown in Fig. 2. The experiment is carried out to scan the object for 40 rotations and 40 linear translations. Pulse waves of the duty cycle of 4.35%, amplitude of 20 V, and pulse width of 2.9e−08 s at a frequency of 1.5 MHz are used to trigger the emitter. In turn, the emitter produces low-power ultrasound waves to scan the object. Low-power ultrasound waves having intensity up to $100 \mathrm{mW}/{\mathrm{cm}}^{2}$ are used for NDT applications as these waves are elastic in nature and cause no harm to the propagating medium^31^. For each rotational angle, 40 linear translations are performed, and data is acquired in each, resulting in a total of 1600 files. Initially, communication to all system modules is established then the data is acquired for each linear translation movement for all the rotation angles. The acquired raw data is saved to the specified directory for further analysis. When the data processing is enabled, acquired data is processed on board to extract meaningful data after each acquisition. This extracted data is used to generate the partial tomograph after each rotation. The performed analysis is visualized on the screen after each rotation. When all the data is processed, final images are projected on the screen and saved in the system. The timing information for each major part of the code is recorded. A video collage (https://www.youtube.com/watch?v=ViV2dGNlxSc) is provided to showcase the complete scanning process using all four languages.

### Performance analysis

The performance analysis of programming language follows as:

#### IDE launch performance

Each IDE is started after a fresh restart of the system, and then it is started several times. The time was recorded until the IDE became responsive. It includes the time taken by the IDE to load background processes for proper functioning. The launch time represents the time taken until the IDE main window appears on the screen, while the response time represents the time until the IDE starts responding to the user commands. It adds up to the overall user experience.

#### Performance indices

Processor utilization(% Processor Time), RAM(Private bytes) usage, IO(Input/Output) Data Bytes/sec, IO Data Operations/sec, IO Read Bytes/sec, IO Read Operations/sec, IO Write Bytes/sec, IO Write Operations/sec are the indices used to analyze the performance^32^. All these eight parameters are recorded while the experiment is running on the system. The codes are executed for both forms with and without signal processing. A performance monitoring tool integrated into the PC’s operating system is used to measure these indices^33^. The I/O processes counter counts all I/O activity generated by the process to include file, network, and device I/Os.

Brief details about the selected indices:

1. **Processor utilization:** Processor Time is a measure of processor utilization by a specific application. It is the percentage of elapsed time that all of the process threads used the processor to execute instructions. Code executed to handle some hardware interrupts and trap conditions are included in this count.
2. **RAM usage:** Private Bytes is the current size, in bytes, of memory that an application has allocated that cannot be shared with other applications.
3. **IO data bytes/sec:** The rate at which the application reads and writes bytes in I/O operations.
4. **IO data operations/sec:** The rate at which the application is issuing read and write I/O operations.
5. **IO read bytes/sec:** The rate at which the application reads bytes from I/O operations.
6. **IO read operations/sec:** The rate at which the application is issuing read I/O operations.
7. **IO write bytes/sec:** The rate at which the application writes bytes to I/O operations.
8. **IO write operations/sec:** The rate at which the application is issuing write I/O operations.

#### Runtime performance

1. In one experiment, data acquisition, read and write timing performance is recorded for 100 data sets with increasing data contents. It helped in analyzing the performance and consistency of the programming languages with an increase in data load. For reading/writing data, the basic .txt format is used for efficiency.
2. In another experiment, timing performance for establishing the connection to the devices and instruments is recorded.
3. The main experiment is carried out to analyze the performance in completing multiple tasks collectively. The time is recorded for multiple sections:
  - for generating graphics of the processed data in each rotation,
  - for measurements for a single rotation with and without signal processing (40 acquisitions in a single rotation, a total of 1600),
  - to complete the experiment.
4. Experiment to test the runtime performance of the languages on different hardware.

## Results and discussion

### IDE launch performance

**Figure 3:**
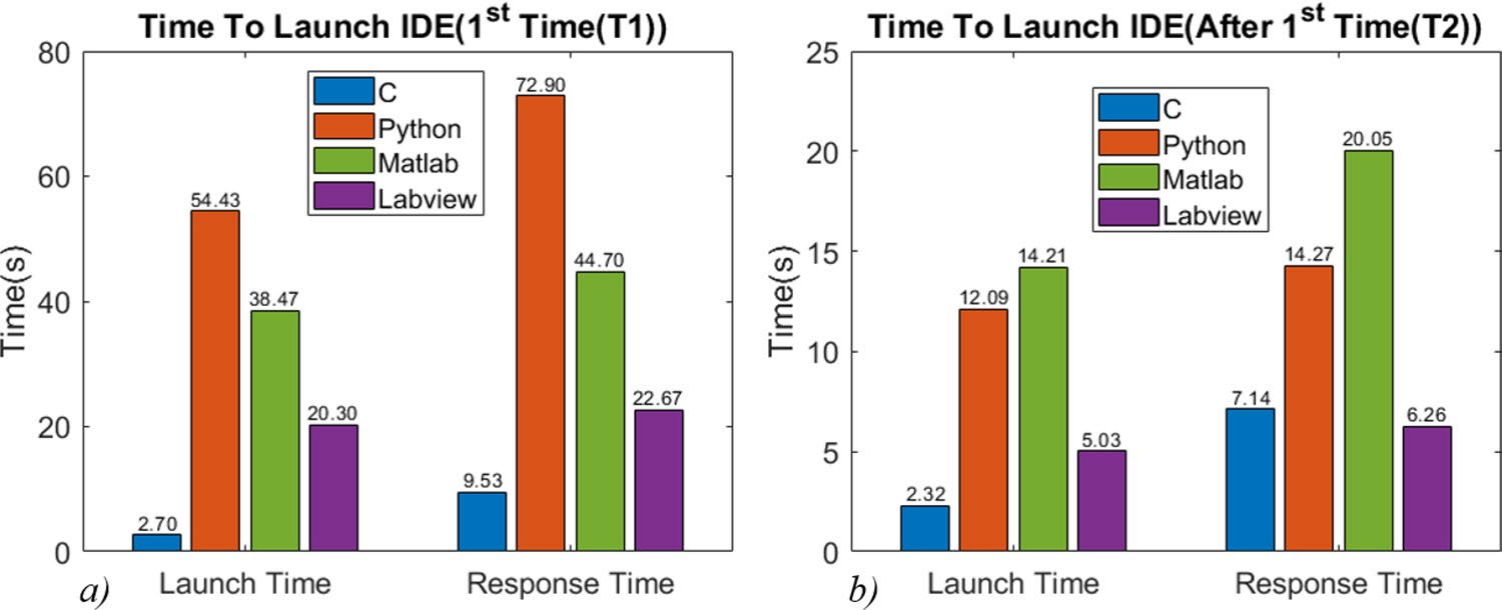
IDE launch performance (**a**) average time to Launch IDE in fresh start (1st time) (T1) and average time until IDE became responsive, (**b**) average time to Launch IDE after 1st time (T2) and average time until IDE became responsive.

The average launch time is measured in two steps: with and without restarting the PC, termed as first launch time (T1) and second launch time (T2), respectively. The exercise to record T1 and T2 is executed six times for each IDE. The mean values are plotted in Fig. 3a and b. The standard deviation values were found to be less than 0.8 s for all the programming languages. In the first launch (T1), the IDE for C language, i.e., VS, took the least launch time, while the Spyder IDE for Python took the maximum time. When these IDEs are started second time (T2) without restarting the PC, VS again showed a faster launch time while LabVIEW showed a faster response time. In every fresh start (T1), each of the IDE took significantly more time than they required when started again (T2), suggesting that several background processes are running (associated with IDE) even after closing it. It may have enhanced the launch performance of the respective IDEs. This behavior is consistently observed for all IDEs.

### Performance analysis

**Figure 4:**
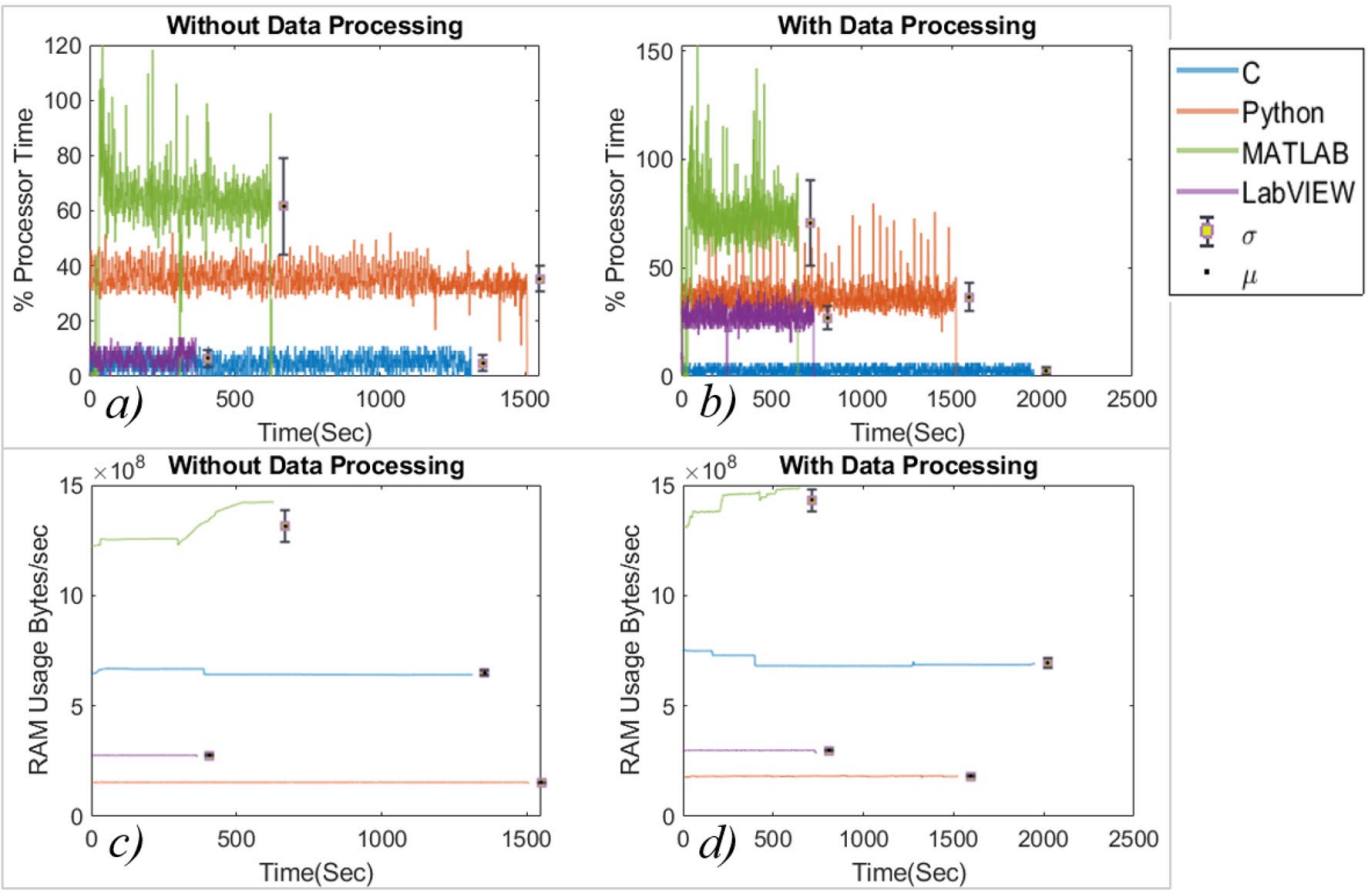
(**a**, **b**) Processor utilization and (**c**, **d**) RAM usage during the experiment.

The plots for the performance analysis of programming languages show the parameter variation during the experiment. The x-axis represents the time taken by the IDE to complete the process. The y-axis represents the respective process. The vertical bar shows the standard deviation (σ), and the black dot indicates the mean (μ) value of the process. The processor and RAM usage with and without data processing are shown in Fig. 4. The C language consumes the least processing power to perform the same analysis while MATLAB consumes the most, as shown in Fig. 4a and b. However, in performing the same processes, Python acquired the least RAM while MATLAB acquired the most, as shown in Fig. 4c and d. Also, processor and RAM usage increased slightly for all languages when the data was also processed during the experiment. The standard deviation was least for C while highest for MATLAB for both processor utilization and RAM usage. In the case of MATLAB, the RAM usage increased as the experiment advanced, suggesting that more memory was acquired to store the working data and the machine codes.

**Figure 5:**
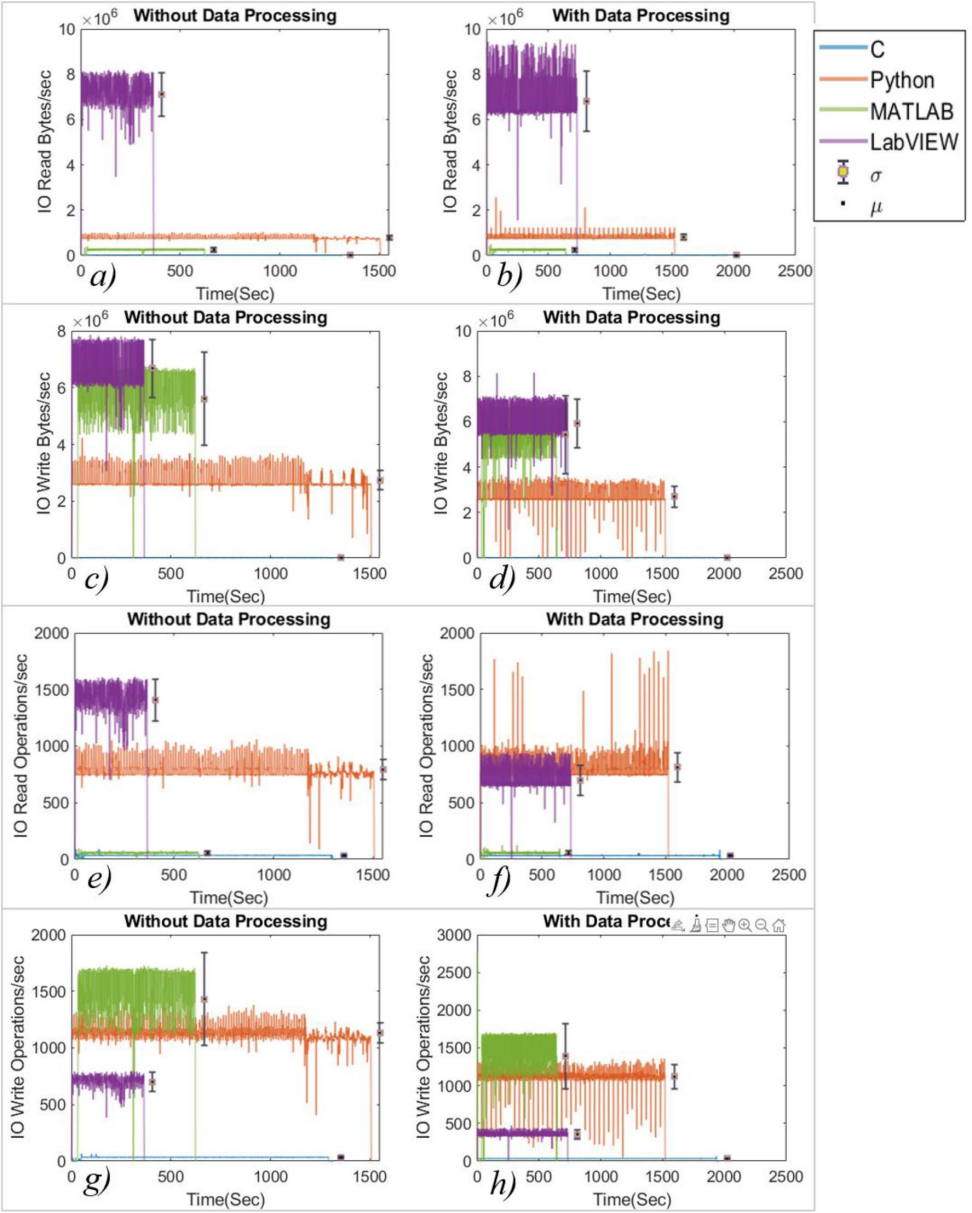
IO read/write bytes and operations executed during the experiment, vertical bar represents the standard deviation (σ) and black dot inside the circle represent the mean (μ) value.

**Figure 6:**
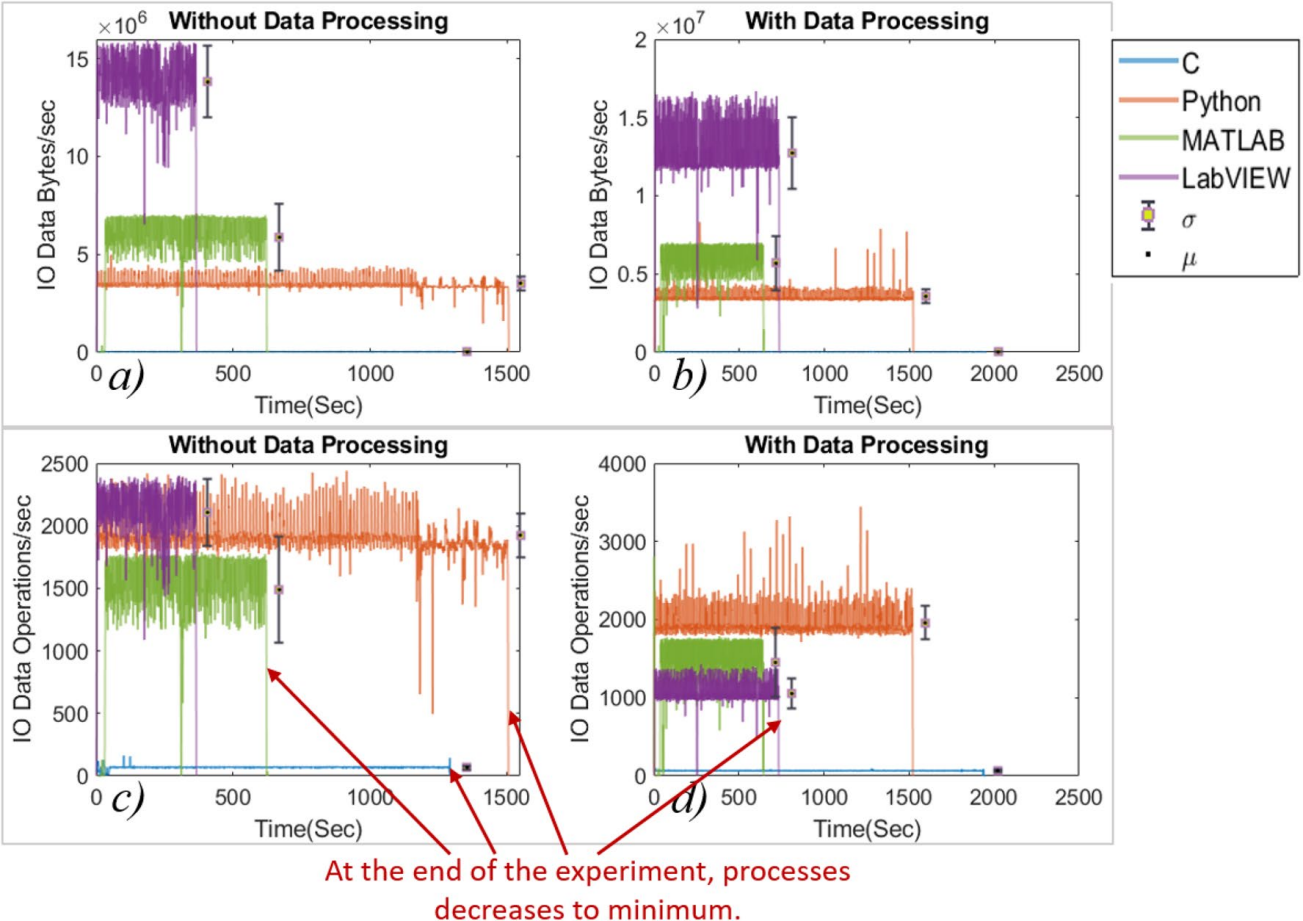
IO data bytes and operations during the experiment.

The IO processes give in-depth information about the processes executed during the experiment. These processes are a measure of the overall memory usage including disk usage that will translate into load on the system due to a particular application. The IO process data contains outliers that affected the visual presentation, a bound at 120% is used to remove such data points. The minimum number of processes/sec are executed for C during the experiment for all the indices in both of the cases (with and without data processing). In the experiment without data processing, LabVIEW executed the higher number of IO read bytes/s, IO write bytes/s, IO read operations/s, IO data bytes/s and IO data operations/s (as shown in Figs. 5a, c, e and Fig. 6a, c, respectively) and MATLAB has executed a higher number of IO write operations (Fig. 5g). However, when the data is processed, LabVIEW executed a higher number of processed only for IO read bytes/s, IO write bytes/s and IO data bytes/s (refer to Fig. 5b, d and Fig. 6b). In comparison, Python executed the highest number of IO read operations/s (Fig. 5f) and IO data operations/s (Fig. 6d), while MATLAB executed the highest number of IO write operations/s as shown in Fig. 5h). The mean values of the performance analysis parameters are tabulated in Table 2. It highlights the best values of performance indices in the green.

The analysis shows that the C performed the least number of IO processes/s, which is also supported by the least processor utilization during both the experiments (with and without data processing). LabVIEW executed a higher number of processes/s in 5/6 IO processes during the experiment without data processing and 3/6 IO processes when the data processing is enabled. As the experiment ends, the number of IO processes along with the processor utilization reduces to a minimum value. However, the RAM usage didn’t decrease even though the processes were reduced to a minimum, suggesting that the IDEs reserve RAM.

The C language has shown the least standard deviation for all processes. Python has shown a higher standard deviation in the experiment with data processing. It is observed that the mean value of processes for programming languages except LabVIEW didn’t change significantly in both cases. It represents that the data processing doesn’t impact the number of IO processes significantly. However, in the case of LabVIEW, the number of IO data, read and write operations/s reduced, which means the operations issue rate is reduced for read and write operations. The languages consumed more time when the experiment was carried out with data processing, as evident from the IO process plots (refer to Figs. 5 and 6) as shown in Fig. 9a and b. In Python, for the experiment with data processing case, it is observed that the processor utilization and the number of IO processes/s bounced several times, matching the number of times the IDE is rendering the graphics, clearly visible in Fig. 5b, d and h. It suggests that Python executes significantly more background processes/s to generate the graphics. Sometimes, during the experiment, the IO processes/s reduce to a minimum value producing a delay in the experiment.

### Runtime performance

**Figure 7:**
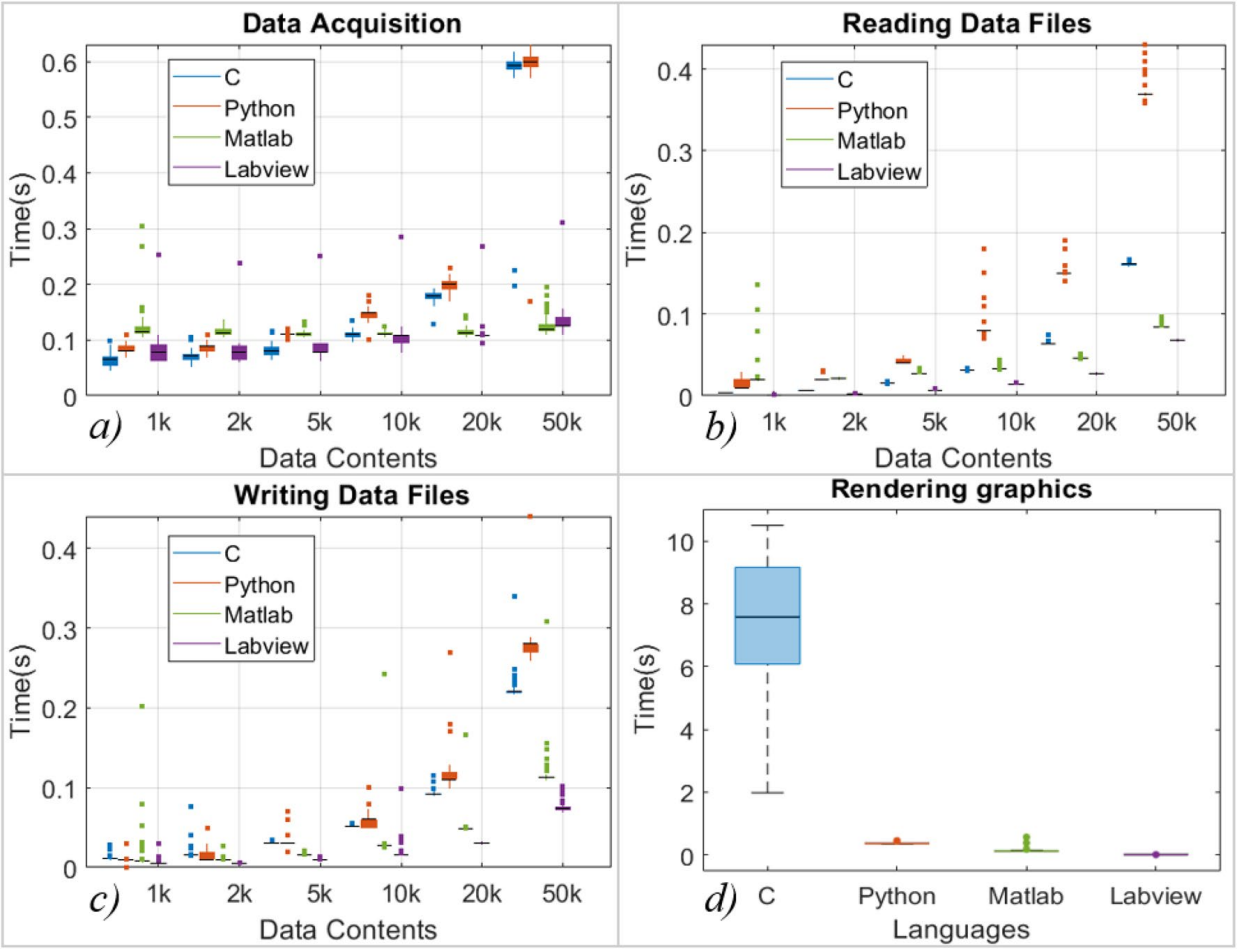
Runtime performance for (**a**) data acquisition, (**b**) reading data files, (**c**) writing data files, (**d**) rendering the graphics during the experiment.

**Figure 8:**
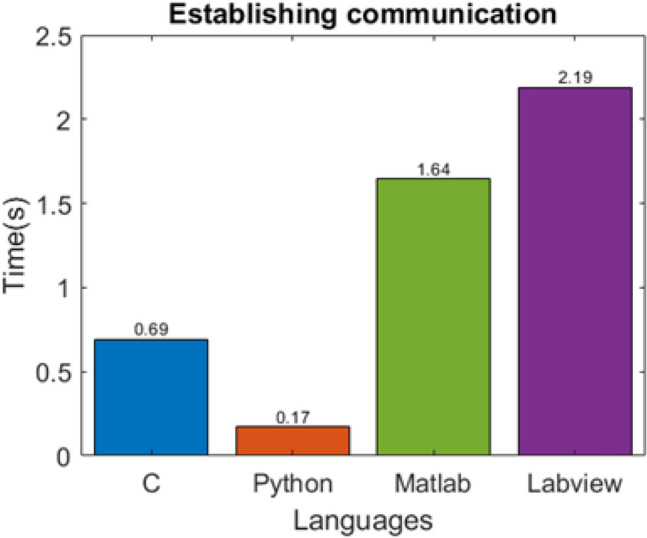
Time consumed to establish communication to the AWG, DSO, and Arduino.

**Figure 9:**
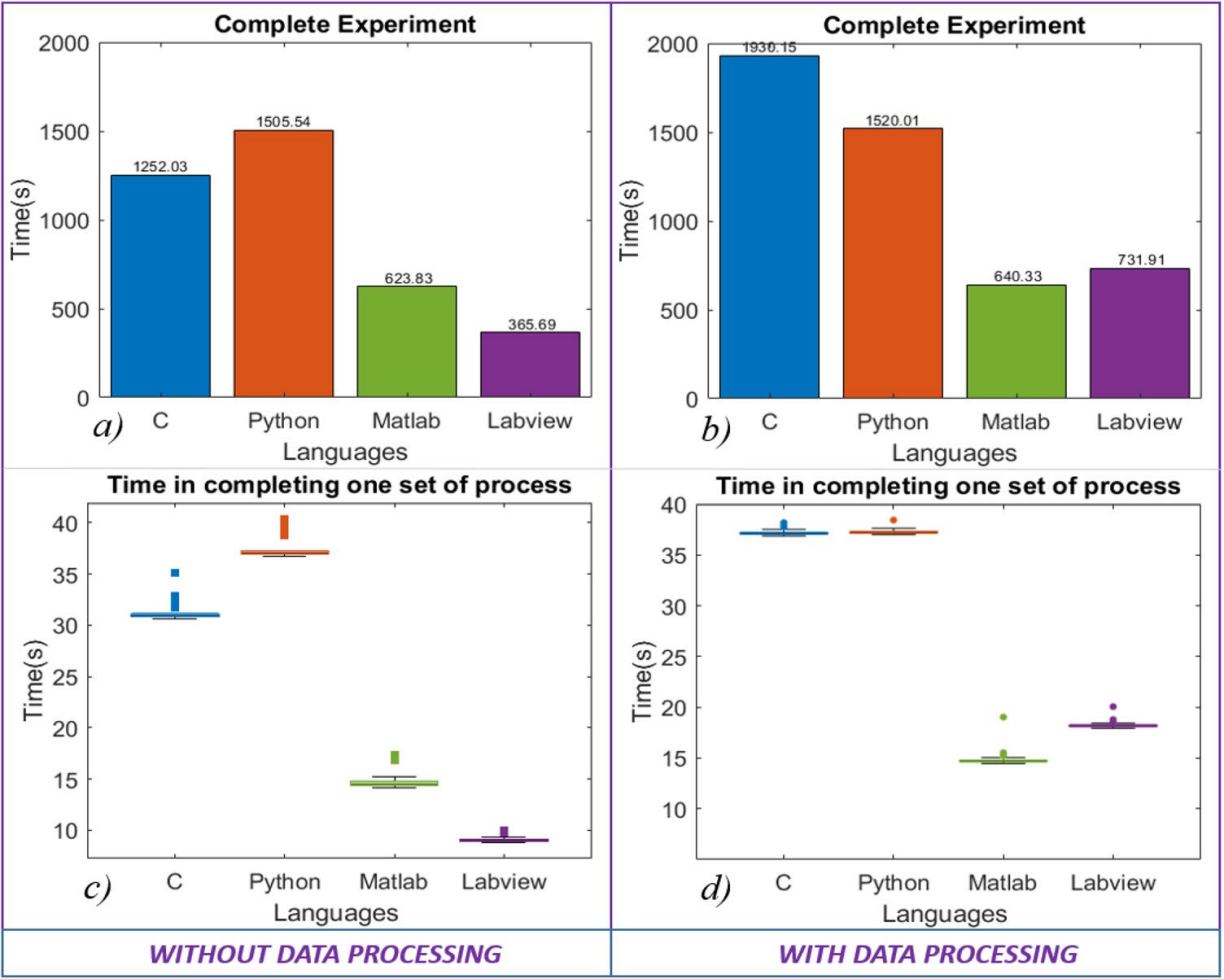
Runtime performance comparison in (**a**) and (**b**) completing the experiment and (**c**) and (**d**) completing one set of process without data processing (**a**) and (**c**) with data processing (**b**) and (**d**).

**Figure 10:**
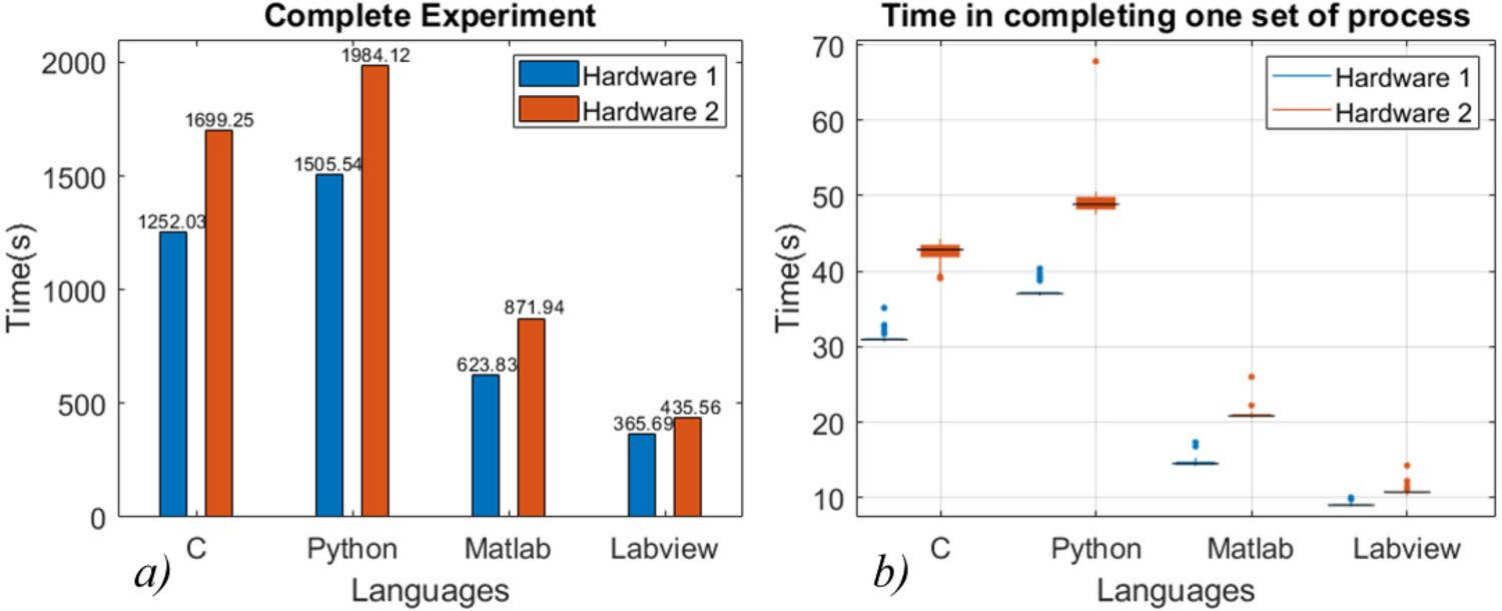
Runtime performance comparison in (**a**) completing the experiment and (**b**) completing one set of process without data processing for hardware 1 and hardware 2*.*

1. Data acquisition, reading and writing speed are measured for 100 data sets with the increase in data contents as shown in Fig. 7a–c. Data sets containing 1 k, 2 k, 5 k, 10 k, 20 k, and 50 k data points are acquired through the DSO, and the same data sets are read and written to .txt files. In data acquisition, the programming language’s performance is mixed; C performed better in acquiring data sets with contents up to 5 k, while LabVIEW performed better for 10 k and 20 k datasets. MATLAB shows better performance in acquiring datasets with higher data contents, whereas C and Python showed a significant drop in performance, as shown in Fig. 7a. Also, MATLAB acquired data with the minimum increase in acquisition time with an increase in the data contents. LabVIEW took the least time to read and write the data files, whereas Python took the most (refer to Figs. 7b and c). Also, the deviation is minimum for LabVIEW and maximum for Python.
2. Establishing communication to the Instruments: The communication is established to the DSO, AWG, and microcontroller using the USB port. Python took the least time while LabVIEW took the most, as shown in Fig. 8.
3. The runtime performance is measured by recording the time at the several parts of the codes,
  - Graphics Rendering: The processed data is visualized after each rotation. After each set, graphics are rendered 40 times during the experiment. The data is represented in the form of the boxplot in Fig. 7d*.* LabVIEW is performing better, while C has shown poor performance in graphics rendering during the experiment.
  - Completing one set of the process: The timing data is recorded 40 times during a single experiment, representing the time taken to acquire the data 40 times and process (when data processing is enabled) it in a single set. A total of 1600 datasets are acquired and saved to the PC. The LabVIEW is faster when the experiment is carried out without data processing, as shown in Fig. 9c. However, MATLAB performed better when the experiment was carried out with data processing, as shown in Fig. 9d. In the second case, C and Python had similar runtime performance, as shown in Fig. 9d. However, all the programming languages showed similar standard deviations.
  - Complete Experiment: LabVIEW has shown better runtime performance in the experiment without data processing, while MATLAB is performing best in another case (refer to Fig. 9a and b). In comparing the runtime performance for both the cases (with and without data processing), the percentage variation in runtime is least for Python and MATLAB.
4. Hardware dependency: The languages performance is compared for two computers having different configurations with specification given in Table 3. The same experiment (without data processing) is carried out on another computer (Hardware 2). The results suggest that the runtime performance of the languages changes with hardware configuration, but the trend/pattern remains same with negligible difference as shown in Fig. 10. However, the standard deviation in C and Python have a higher value in case of hardware 2 as shown in Fig. 10b). The observations suggests that the execution time of certain algorithm when tested using various programming language may vary according to the PC configuration.

## Discussion

The analysis was performed without optimizing codes for any particular language (i.e., using the same coding style wherever possible). Analysis provides a scientific methodology to analyze the performance of programming language from a given set of tools and resources. Based on the analysis, one can choose the best language according to the application requirement. In addition, library richness, online forums, user experience, and code length play an important role in the selection of a programming language.

In the presented analysis, the C language’s poor performance in graphics visualization may be due to interfacing “Gnuplot” into the C code. The Gnuplot window took much time to visualize. Working with C language requires advanced technical knowledge and experience to develop coding skills. The C language provides some advantages over others such as low-level control in memory management. It can make your applications run faster or become slower over time if one doesn’t keep track of memory flow. Running the executable over time with memory leaks results in significant memory leaks that can potentially lead to application crashes. It may result in an inefficient code in terms of ram utilization, memory utilization and timing performance. It will result in larger inefficiencies while dealing with larger datasets. The tracking of performance parameters can provide information about such issues. LabVIEW inherently optimizes the codes to execute in parallel. So, the codes are written in such a way that the code will be executed step by step. Moreover, it is difficult in developing large codes in GUI-based languages because of the problem in keeping track of the previous codes. However, subVI can help a lot in such conditions, but even it is difficult to develop complex codes because of the limited view into the working window. Working with Python and MATLAB offers some advantages as they are supported by a strong academic and research community and online resources.

Conventionally, the selection of a programming language primarily depends on the convenience and application without testing the performance. The cost may also be a significant factor in selecting a particular language. The MATLAB® and LabVIEW™ are proprietary and closed-source programming environments developed by MathWorks and National Instrument (NI), respectively. A license is required to be purchased, which may be expensive for some users. License prices are subject to vary according to country, number of licences and type (personal use, commercial use, or academic institutional usage), duration of use, version, and integrated toolbox/modules. MATLAB standard license starts from 70,000 INR/year while the LabVIEW full license starts from 143,700.00 INR/year^34,35^. On the other hand, C and Python are open-source software and available at no cost.

## Conclusions

A comparative analysis of four programming languages: C, Python, MATLAB, and LabVIEW, if presented. Apart from the runtime analysis, two experiments for each language were performed to compare the programming language’s performance in terms of various performance indices. The first experiment was performed without on-board data processing. While the other was performed with data processing.

IDE for the C language showed a faster launch and response time. Also, the C language performed better in processor optimization and IO processes. The launch performance has significance. If the probability of an operating system or IDE crash (for example, due to some programming fault, high load) is relatively high and the time is limited, C would be preferable.

The C language is the best choice for the system or embedded system with limited processing power and memory. Python performed better in establishing communication with the instruments and in RAM usage. It would be preferable when more instruments or devices are required to be interfaced.

In runtime analysis,

1. In data acquisition, C language is performing better for data contents up to 5 k, LabVIEW for contents 10–20 k, while MATLAB is performing better for higher data contents. However, the performance of all the languages is comparable for the acquisition of datasets having contents up to 10 k.
2. In reading/writing data files, LabVIEW is performing better. Also, LabVIEW has shown consistent runtime performance. Again, the performance of all the languages is comparable for data contents up to 5 k.
3. LabVIEW execution was faster for the experiment without data processing, while MATLAB was faster for the other case.
4. In graphics rendering, LabVIEW is performing better. However, the standard deviation is negligible for Python, MATLAB and LabVIEW.
5. The runtime performance of the languages may vary according to the different hardware configurations.

LabVIEW performed better in real-time control of the electromechanical assembly and synchronous data acquisition, and Python performed better runtime efficiency in sensors, and instruments integration, while MATLAB is faster in the simultaneous raw data processing. The C language performed better in optimizing resources, indicating suitability if resources are relatively inferior in processing power and storage. Sometimes, the runtime efficiency provided by a language can be countered by the time taken to write the codes. The optimal programming language implementation is an essential requirement of a data intensive and complex system. This will enhance the performance and efficiency significantly. For even better performance, multiple optimal language can be interfaced by writing integration code to perform specific operation in an efficient language. So, the selection of programming languages based on tested performance analysis tools will result in better efficiency.

## Data Availability

A movie (https://www.youtube.com/watch?v=FuTamEnlWUk) is provided to show the relative performance of all the programming languages. The codes used in the analysis can be made available on request by contacting the corresponding author.
